# Strong Stability and Host Specific Bacterial Community in Faeces of Ponies

**DOI:** 10.1371/journal.pone.0075079

**Published:** 2013-09-11

**Authors:** Tina M. Blackmore, Alex Dugdale, Caroline McG. Argo, Gemma Curtis, Eric Pinloche, Pat A. Harris, Hilary J. Worgan, Susan E. Girdwood, Kirsty Dougal, C. Jamie Newbold, Neil R. McEwan

**Affiliations:** 1 Institute of Biological, Environmental and Rural Sciences, Aberystwyth University, Aberystwyth, Ceredigion, Wales; 2 Division of Equine Science, Department of Veterinary Clinical Science, University of Liverpool, Leahurst, South Wirral, England; 3 Equine Studies Group, WALTHAM Centre For Pet Nutrition, Waltham-On-The-Wolds, Leicestershire, England; University Paris South, France

## Abstract

The horse, as a hindgut fermenter, is reliant on its intestinal bacterial population for efficient diet utilisation. However, sudden disturbance of this population can result in severe colic or laminitis, both of which may require euthanasia. This study therefore aimed to determine the temporal stability of the bacterial population of faecal samples from six ponies maintained on a formulated high fibre diet. Bacterial 16S *rRNA* terminal restriction fragment length polymorphism (TRFLP) analyses of 10 faecal samples collected from 6 ponies at regular intervals over 72 hour trial periods identified a significant pony-specific profile (P<0.001) with strong stability. Within each pony, a significantly different population was found after 11 weeks on the same diet (P<0.001) and with greater intra-individual similarity. Total short chain fatty acid (SCFA) concentration increased in all ponies, but other changes (such as bacterial population diversity measures, individual major SCFA concentration) were significant and dependent on the individual. This study is the first to report the extent of stability of microbes resident in the intestinal tract as represented with such depth and frequency of faecal sampling. In doing so, this provides a baseline from which future trials can be planned and the extent to which results may be interpreted.

## Introduction

As with other mammals, the horse provides a complex, but generally symbiotic, environment for the microbes in its digestive tract [1]. These microbial communities provide an integral mechanism where dietary complex carbohydrates can be degraded into a form which can be absorbed across the digestive tract wall, making nutrients available for use to the host [1]. This is particularly notable in herbivores as mammals lack the enzymes necessary to fully degrade the complex plant substances present in their diet. The microbial population secrete a range of enzymes, which are able to degrade and initiate fermentation of polysaccharides to short chain fatty acids (SCFA), which in turn can be absorbed and utilised as energy sources by the host animal. In return, the animal provides the microbes with a homeostatic environment and the initial physical disruption of the fibrous material, via mastication, forming a symbiotic association between the microbes and their host.

An increasing number of studies have begun to explore the diversity and complexity of the population that reside in the horse via next generation sequencing (NGS) [2]–[5]. As initial studies using Sanger sequencing methods suggested, the majority of the bacterial species amplified are novel with 89% and 96% of sequences with <97% identity to any sequences described previously [6]–[8]. However, due to the associated cost of NGS, the number of samples or horses is a restricting factor on determining how representative a single sample may be or how the population structure varies under uniform normal conditions.

Studies to date have relied on reporting findings from a single time point, or limited repeated sampling following a dietary change (commonly a rapid and extreme change) or disease induction. A number of diseases are associated with rapid changes in diet and associated sudden shifts in the microbial population in the hindgut, such as acidosis, colic and laminitis [9]–[11], which can be sufficiently severe to require euthanasia. Whilst this work is essential to the management and welfare of animals along with elucidating the pathology of a disease, there is a lack of knowledge on the normal stability or fluctuations in the hindgut of horses maintained on a uniform diet.

The current work aims to describe and investigate the temporal stability of the faecal bacterial population of ponies maintained under constant conditions (including a uniform diet) over 2 trial periods 11 weeks apart. In addition to this, the presence or absence of host specific bacterial populations can be identified.

## Results and Discussion

### General observations

No ponies showed any signs of colic nor diarrhoea during the study. During TP1 and TP2, all defecations throughout the 72h were collected. Significant differences in the number of defecations were recorded between ponies (P<0.05), averaging 53 (SD 18.8), but ranging from 18 to 80 defecations over the 72 h sampling periods. This was higher than the 5 to 10 defecations per day previously reported in healthy horses [12]. A TP effect was also found, with a reduction in the number of defecations from TP1 to TP2 from an average of 19 defecations per day to 16, respectively (P<0.001). Passage rate has been shown to be influenced by diet, meal time, hydration status, exercise amongst other factors [13].

### Comparison of bacterial population

**Figure 1:**
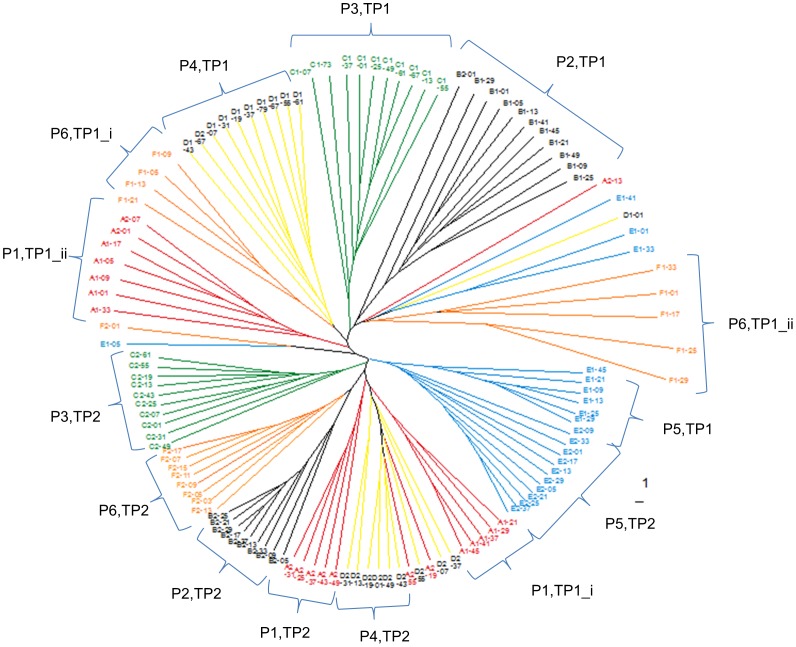
Dendrogram of Hamming Distances (binary abundance) from TRFLP data. 10 samples per pony (n = 6) per trial period (1, 2) are represented. Dendrogram coloured by pony and samples clustering by pony and trial period are labelled (eg P1,TP1 represents pony 1 in TP1). i, ii denote first and second clusters within pony and trial period.

Ten bacterial population TRFLP profiles from all 6 ponies and both TP were initially investigated using binary data with Hamming Distances, in order to compare the differences in the presence or absence of TRF peaks between samples (Figure 1). A temporal change was apparent with the segregation of samples collected in TP2 relative to those from TP1, with the exception of pony 5. The similarity of bacterial populations was observed to be greater in TP2 than TP1, as shown by the reduced branch lengths. Significant differences between trial periods were identified (PERMANOVA; Pseudo-F  =  17.571, P = 0.0001) with greater similarity of bacterial populations (less average distance) between ponies in TP2 than TP1. Pony-specific sample clustering was also observed showing significant differences between ponies (PERMANOVA; Pseudo-F  =  11.127, P = 0.0001). Within a pony, significant differences between TP were found (P<0.002), with greater similarity of bacterial populations between defecations (reduced average distance) observed in TP2 for 4 ponies (ponies 2-4,6).

**Figure 2:**
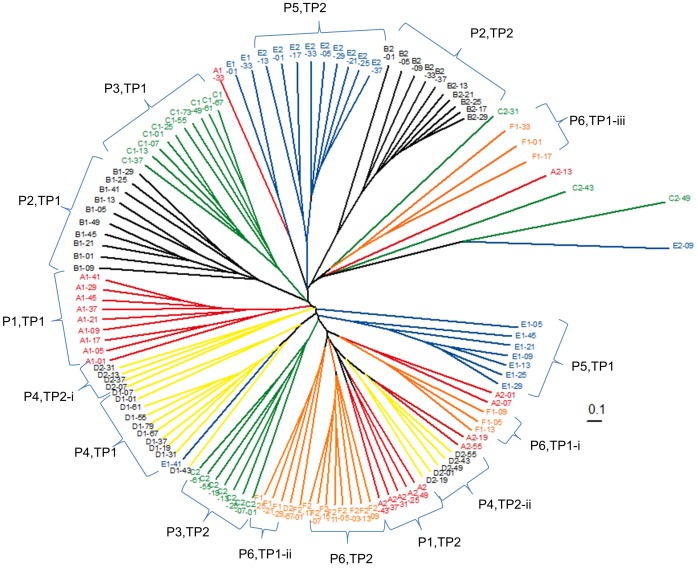
Dendrogram of Manhattan Distances (relative abundance) from TRFLP data. 10 samples per pony (n = 6) per trial period (1, 2) are represented. Dendrogram coloured by pony and samples clustering by pony and trial period are labelled (eg P1,TP1 represents pony 1 in TP1). i, ii denote first and second clusters within pony and trial period.

The similarities between bacterial populations were then explored with the incorporation of relative peak height data and the use of Manhattan Distances (Figure 2). As with the binary data, significant differences were found with the incorporation of the relative abundance of each TRF peak between trial periods (PERMANOVA; Pseudo-F  =  17.538, P = 0.0001), between ponies (PERMANOVA; Pseudo-F  =  11.39, P = 0.0001) and interaction between the groups. Bacterial populations observed at TP2 were found to be more similar, both between and within ponies, than at TP1, as shown by reduced average branch distance.

**Figure 3:**
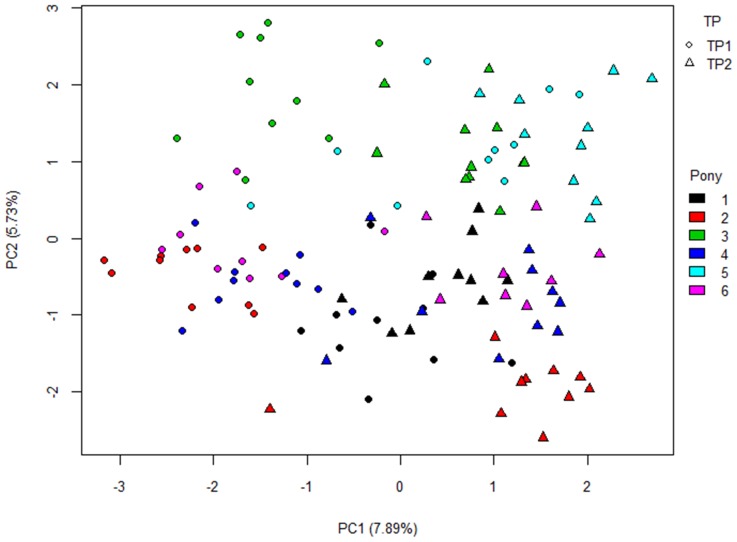
Principal component analysis plot for binary data. Split of data from trial period (TP) 1 to TP2 observed on PC1. Each pony denoted by different colour. TP1 marked by circles, TP2 by triangles.

Data were also analysed using Principal Component Analysis (PCA) (Figure 3) and Discriminant Function Analysis (DFA) (data not shown). These plots reflected the clustering pattern observed in the dendrograms for binary and relative data, showing evidence of clustering in a pony-specific manner (and also the temporal split in the binary data; Figure 1).

Population diversity is described in terms of richness, or the number of different species present, and evenness, or the equal abundance of each species. As a proxy for diversity of bacterial populations using TRFLP, the number of TRF peaks and their relative abundance are used as a guide. Comparison of population richness, as determined by the number of TRF peaks, identified highly significant differences between ponies and TP for each restriction enzyme (P<0.05) (Table 1). Evenness, calculated using Shannon Equitability (E_H_), is a measure of the equality of species abundance in a population, where 1 is equal presence. Shannon Diversity Index (H’) accounts for both the abundance and evenness. A similar overall pattern to richness was seen for H’ and E_H_ (P<0.01), with the exception of no significant change in E_H_ found from TP1 to TP2 for *Msp*I. Both *Msp*I and *Hae*III showed reductions in the number of TRF peaks detected between TP1 and TP2 (P<0.05), while *Scr*FI showed no differences in TRF number between TP1 and TP2 in 4/6 ponies, with an increase in TRF number in the remaining 2 ponies. This reduction in TRF number indicates a reduced richness. Each restriction enzyme (RE) has been analysed separately for measures of diversity due to potential differences in the frequency of a specific recognition site across the bacteria present in that population. A reduction in E_H_ and H’ was found for 4/6 ponies with *Hae*III. Coupled with the reduction in the number of TRF between TP1 and TP2 for this RE, this suggests a change in the population structure to one with a more dominant presence of a limited number of TRF (approximating to species). The changes observed for the other two RE are less clear, with increases (and decreases) in TRF number and evenness detected in some ponies. Results from all 3 RE taken together conclusively support a change in the population structure (richness and evenness) between the 2 trial periods.

**Table 1:** Bacterial population diversity indicators: number of TRF, Shannon Diversity Indices and Evenness.

		No. of TRF					Shannon Diversity Indices					Shannon Equitability				
	Pony	TP1		TP2		P^1^	TP1		TP2		P^1^	TP1		TP2		P^1^
		Mean	SE	Mean	SE		Mean	SE	Mean	SE		Mean	SE	Mean	SE	
MspI	1	22.2^a^	0.98	22.6^ b^	0.99	ns	2.81^ ab^	0.037	2.81^ b^	0.048	ns	0.910^b^	0.0035	0.905^ab^	0.0047	ns
	2	23.4^ ab^	0.56	18.6^ ab^	1.37	**	2.87^ abc^	0.022	2.60^ ab^	0.065	***	0.910^ b^	0.0028	0.895^a^	0.0031	**
	3	24.8^ ab^	2.00	17.2^ a^	1.49	**	2.94^ bc^	0.059	2.50^ a^	0.110	**	0.925^ b^	0.0054	0.892^a^	0.0104	*
	4	22.0^ a^	1.10	20.9^ ab^	0.99	ns	2.73^ a^	0.053	2.74^ ab^	0.044	ns	0.887^ a^	0.0041	0.904^ab^	0.0031	**
	5	20.0^ a^	0.82	21.2^ ab^	0.59	ns	2.71^ a^	0.034	2.81^ b^	0.028	*	0.907^ b^	0.0045	0.921^b^	0.0026	*
	6	27.8^ b^	1.53	21.3^ ab^	0.88	**	3.02^ c^	0.050	2.78^ b^	0.034	**	0.911^ b^	0.0037	0.911^ab^	0.0025	ns
	P^2^ value	**		*			***		**			***		**		
ScrFI	1	13.5^ b^	0.72	11.0^ ab^	0.95	ns	2.15^ b^	0.066	1.82^ a^	0.077	**	0.828^ c^	0.0102	0.768^a^	0.0074	***
	2	11.6^ ab^	0.70	14.8^ c^	0.33	**	1.87^ ab^	0.048	2.31^ bc^	0.012	***	0.768^ ab^	0.0093	0.858^c^	0.0031	***
	3	9.3^ a^	0.78	10.6^ ab^	0.93	ns	1.72^ a^	0.098	1.95^ ab^	0.089	ns	0.779^ abc^	0.0226	0.847^c^	0.0191	*
	4	11.2^ ab^	0.59	13.3^ bc^	0.84	ns	1.99^ ab^	0.064	2.16^ bc^	0.073	ns	0.827^ bc^	0.0082	0.840^bc^	0.0082	ns
	5	11.2^ ab^	0.77	9.5^ a^	0.79	ns	1.96^ ab^	0.086	1.74^ a^	0.066	*	0.820^ bc^	0.0171	0.791^ab^	0.0179	ns
	6	9.9^ a^	0.54	13.4^ bc^	0.87	**	1.69^ a^	0.042	2.01^ abc^	0.07	**	0.743^ a^	0.0089	0.776^a^	0.0124	*
	P^2^ value	**		***			***		***			***		***		
HaeIII	1	17.5^ a^	0.76	17.9	1.35	ns	2.49^ a^	0.053	2.53^ b^	0.079	ns	0.871^ a^	0.0073	0.886^b^	0.0070	ns
	2	23.1^ a^	1.18	12.7	1.65	***	2.80^ b^	0.049	2.07^ a^	0.127	***	0.895^ ab^	0.0043	0.831^a^	0.0155	***
	3	19.6^ a^	1.02	15.3	0.87	**	2.62^ ab^	0.053	2.36^ ab^	0.063	**	0.885^ a^	0.0048	0.868^ab^	0.0062	*
	4	29.1^ b^	1.74	18.3	0.83	***	3.08^ c^	0.052	2.61^ b^	0.047	***	0.919^ bc^	0.0036	0.900^b^	0.0037	**
	5	23.0^ a^	1.51	17.4	1.90	*	2.87^ bc^	0.061	2.45^ ab^	0.112	**	0.922^ c^	0.0049	0.874^b^	0.0081	***
	6	22.0^ a^	1.60	17.4	1.38	*	2.71^ ab^	0.082	2.46^ ab^	0.089	ns	0.882^ a^	0.0085	0.865^ab^	0.0107	ns
	P^2^ value	***		ns			***		**			***		***		

Average and standard error (SE) for each pony within trial period and for each restriction enzyme shown. P^1^ values identify significant changes between TP within pony and RE. P^2^ values identify significant differences between ponies, within TP and RE. ns – not significant, * P<0.05; ** P<0.01; *** P<0.001.

The Shannon Diversity Index values are slightly lower than previously reported value of 3.46 from TRFLP analysis of horses faeces [3]. This may reflect differences between experiment comparison (although the method used is comparable) or differences in diet (high fibre) and the uniformity of its intake. Other studies investigating bacterial population composition in faeces that have calculated H’ have reported much greater values (exceeding 5) [2], [4]. However, these studies have used 454 NGS, which will provide a much greater depth and breadth of species identification in contrast to TRFLP. The bacterial evenness (a measure of uniformity of species abundance in the population and therefore independent of detection sensitivity) of these populations was found to be similar in this study, compared to others [2]. Diversity values are, therefore, most informative when used as a comparison within a study or using a similar method. It is unknown whether the reduction of TRF number, as a proxy for the reduction in richness, represents a loss of bacterial species from the population, or whether numbers were reduced to below the detection threshold and could potentially recover.

Culture independent techniques of bacterial population investigation are important in order to estimate the range and composition of a microbial community at a point in time. TRFLP is a PCR-based community fingerprinting method that allows analysis of the structure (in terms of abundance and presence) of a population [14]. The technique is reproducible and comparable across multiple samples [14]. These advantages of reproducibility are important in contrast to other commonly used microbial community profiling methods, e.g. denaturing gradient gel electrophoresis (DGGE) or temperature gradient gel electrophoresis (TGGE). Comparison of DGGE/TGGE samples and populations is only accurate within a single gel, thus severely restricting the number of samples that can be compared at any one time. Whilst TRFLP does not provide the same depth of information on a bacterial population as found in next generation sequencing, such as the defining the composition to a species level, its accuracy, reproducibility and cost effectiveness are important subsequent considerations to the main priority of the question posed. By providing bacterial population profiling of such a large number of samples in order to ascertain the stability of the population between and within individuals, TRFLP is an ideal choice.

### Comparison of metabolomes

In order to integrate differences observed in the bacterial population with functional activity, two methods of metabolite screening were used. The primary activity of the bacterial population that reside in the hindgut is fermentation, the major products of which are short chain fatty acids (SCFA) [1]. These can be absorbed across the gut mucosa and utilised by the horse as a significant energy resource [1]. Significant differences in the total (major) SCFA detected in the faeces were found between ponies during TP1 (P<0.01; Table 2), whereas during TP2, no significant inter-animal variation between SCFA concentrations was observed. Overall, average SCFA concentrations were higher in TP2 than TP1. This pattern was also found for acetate concentration. For the other major SCFA, butyrate, propionate and valerate, significant differences in concentration were found between ponies for both trial periods. A large increase in propionate was detected in ponies 2 and 6, which contrasted to the other ponies where an observed decrease in concentration was found, although not significant. The ratio of SCFA produced was also tested and significant changes identified (Table S1). With the exception of pony 5, the proportion of acetate remained unchanged between TP within each pony, but the relative abundance of the other SCFA altered. No generalised change in the SCFA concentration or ratio between TP for all ponies can be drawn, with each pony behaving uniquely (Table 1 and S1).

**Table 2:** Concentration (mM) of total and each major SCFA, for each pony and trial period.

	Pony		1	2	3	4	5	6	P^2^ value
Total SCFA	TP1	Mean	96.01^ab^	72.57^bc^	105.71^a^	62.13^c^	72.27^bc^	76.18^abc^	**
		SE	7.950	5.473	6.428	6.413	6.132	8.818	
	TP2	Mean	93.38	99.68	107.08	78.78	99.86	104.71	ns
		SE	9.719	6.453	7.295	4.757	6.867	7.627	
		P^1^ value	ns	**	ns	ns	**	*	
Acetate	TP1	Mean	77.85^ab^	58.58^bc^	84.43^a^	51.04^c^	56.39^bc^	61.35^abc^	***
		SE	6.718	4.339	5.098	5.592	4.875	6.982	
	TP2	Mean	75.96	79.86	86.84	66.57	83.52	85.24	ns
		SE	7.352	5.246	6.428	4.649	5.564	6.622	
		P^1^ value	ns	**	Ns	*	**	*	
Propionate	TP1	Mean	13.40^ a^	9.79^ ab^	13.11^ a^	8.24^ b^	10.70^ ab^	9.33^ ab^	**
		SE	1.031	0.792	0.865	0.696	1.241	1.387	
	TP2	Mean	11.03^ ab^	15.34^ a^	12.86^ ab^	8.48^ b^	10.59^ ab^	12.67^ ab^	***
		SE	1.862	1.096	0.799	0.553	0.983	0.673	
		P^1^ value	ns	***	ns	ns	ns	*	
Butyrate	TP1	Mean	4.06^ b^	3.44^ b^	5.99^ a^	2.67^ b^	3.96^ b^	4.12^ b^	***
		SE	0.327	0.322	0.424	0.186	0.346	0.576	
	TP2	Mean	4.98^ ab^	3.86^ ab^	5.67^ a^	3.10^ b^	4.28^ ab^	5.47^ a^	**
		SE	0.69	0.284	0.418	0.214	0.614	0.493	
		P^1^ value	ns	ns	ns	ns	ns	*	
Valerate	TP1	Mean	0.70^ cd^	0.76^ cd^	2.18^ a^	0.18^ d^	1.22^ bc^	1.39^ b^	***
		SE	0.103	0.119	0.157	0.097	0.156	0.243	
	TP2	Mean	1.41^ ab^	0.62^ b^	1.72^ a^	0.63^ b^	1.47^ ab^	1.32^ ab^	**
		SE	0.306	0.078	0.14	0.091	0.378	0.294	
		P^1^ value	*	ns	*	**	ns	ns	

P^1^ values identify significant changes between TP within pony and each SCFA. P^2^ values identify significant differences between ponies, within TP and each SCFA. ns – not significant, * P<0.05; ** P<0.01; *** P<0.001.

The total SCFA concentrations in the faeces were towards the higher values of the range previously reported in the small colon or faeces of the horse, but values not dissimilar to those recorded in the colon (Table 2) [3], [15], [16]. However, higher concentrations of SCFA are reported in the colon and caecum, and can be greater in horses on a high fibre diet [17]. While total bacterial numbers may not increase on a high fibre diet, the number of cellulolytic bacteria have been found to be 100-fold greater with a total fibre diet (100% hay) to ones including 30 or 50% barley [18]. Increased concentrations in SCFA measured in the faeces at TP2 may be a reflection of the *ad libitum* availability of fermentable fibre in the diet. This, coupled with limited exercise and energy demand on the animal, may therefore lead to an excess of SCFA production.

**Figure 4:**
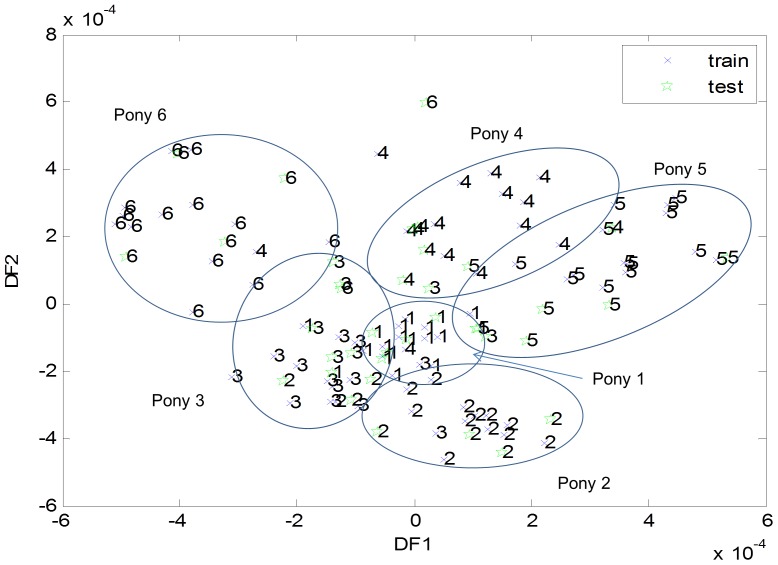
Cross validated discriminant function analysis plot for relative FTIR data by pony (both trial periods). Trained data used in the original validation process are denoted by a cross, whilst test data are denoted by a star. Between the two DF values 99.66% of the total variance was described.

Fourier Transform Infrared (FTIR) spectroscopy has been used to compare metabolites from a range of different environments, including that of differing sections of the horse’s digestive tract and sheep faeces [3], [19]. This technique showed a general trend towards host-specific clustering of metabolites when investigated by DFA (Figure 4), but neither pony-specific nor temporal effects were seen for PCA (data not shown). The loss of strong host specificity using FTIR analysis may reflect a comparable functionality of the bacterial populations (i.e. similar metabolome), but with it being provided by different bacterial species.

### General Discussion

It is clear that a strong pony-specific effect existed in the individuals used in this study and the metagenomic and metabolomic techniques employed. Host specific effects on bacterial populations have been commented on before as showing large inter-horse variations [3], [5], [20]. Similar observations have been described in cattle [21], [22], prompting a specific investigation of inter-rumen population composition and similarity [23].

Inter-individual variation in the horse digestive tract has been observed before, although not specifically tested [10], [20], [24]. A previous study that used T-RFLP only showed a limited grouping of samples by horse, however, there was reduced sampling frequency (weekly), coupled with changes in diet and the horses were in race training [25]. The latter variable (exercise) has been shown to alter the passage rate and bacterial population in ponies [13], [26]. Schoster et al [27] compared different sections of the intestinal tract from 6 horses of the same breed and from the same farm, to another 4 horses. They found significant differences between the 2 groups in the duodenal and caecal regions, with no difference in other sections, including the faeces. Comparison of the faeces between horses of the same breed had an average Dice similarity of 0.54, suggesting limited similarity [27]. A significant difference between animals was found using TRFLP on different sections on the equine digestive tract [3], in addition to a Thoroughbred/New Forest pony effect. In our study, only ponies of a similar type (Welsh Mountain ponies) were used, thereby simultaneously minimising both the genetic variability and the dietary variation.

Bacteria have been isolated from the whole digestive tract of the horse, but as a hindgut fermenter the greatest number are resident in the caecum and colon [15], [28]. The importance of this hindgut population to the horse is shown by the anatomical adaption to fermentation by enlargement of the caecum and large colon to a capacity of around 55L and 80L, respectively, in a 500 kg horse [29]. The size and composition of the microbial population changes through the tract [3], [15], [27]. The degree to which faeces are representative of the intestinal tract of the horses has been studied [3], [27], [30]. These studies have used bacterial profiling methodologies (TRFLP [3], [27] and automated ribosomal intergenic spacer analyses [30]) to identify that the faeces are similar to the latter sections of the large colon, but dissimilar to the caecum. The point or gradient over which this change occurs should be subject to further work. Immediate sampling of voided faeces allows repeated non-invasive sampling of any horse and any number of horses. Faeces, therefore, provides a useful vector by which the distal hindgut may be sampled.

Following the 11 week sampling period interval, strong pony-specific effects remained. Stability and host-specific populations have been observed at greater inter-sampling period times in humans. Stability of the population composition was observed after 6 months in 2 people [31], and a longitudinal study also demonstrated that a subject-specific microbiota was present at intervals less than 1 year and after 10 years [32]. Individual specific clustering was also found in 10 adults in faecal sampling over 2 months using the microarray-based approach of the HITchip [33]. In addition to which, greater similarity was detected using this greater depth/accuracy/robustness when comparing DGGE results of the same samples, suggesting the large similarity within an individual as detected by some methods may actually be greater than initially apparent.

Comparable results to our study have also been identified in cattle over shorter sampling intervals [21], [22]. Repeated individual bovine rumen sampling over 48h found differences between 2 cows [21] and over 12h in 3 cows [22]. The latter study found 93% population similarity within a cow over time but only 85% between cows. A limited extension to these results showed over 90% similarity of weekly sampling from a cow for 4 weeks [22].

A few general changes observed between the 2 sampling periods were common to all ponies, such as increased bacterial population stability and increased total SCFA concentration. However, most changes were dependent on the individual. Significant intra-pony increases were recorded between trial periods, but for the same parameter, a significant decrease was also observed in a different pony (Table 1 and 2). Our study offers insight into the potential problems with using ponies as replicates of a treatment effect. If the data were considered as a group, no change may have been found, however, the individual responses were in fact significant. This may have no real implication at the level of the entire herd, although it should be considered that in some individuals a significant change has occurred. This concern is highlighted by 5 horses used in trial to investigate the microbial response to oligofructose dosing to induce laminitis [10]. Horse specific changes of differing temporal responses were seen in total bacterial and species specific population, as well as lactate, SCFAs and fructo-oligosaccharides over 36 hours. The difficulty in forming a general summary between the trial periods in our study highlights the potential impact on future experimental designs. This could be a large effect if sample or animal numbers are low. This is a persistent limitation of animal trials due to cost/space. However, when trying to detect a small change it may be worth considering the effect for each animal, or reassessing the cost/benefit ratio.

The stability of bacterial populations over 72h within a pony was very high and reproducible in both trial periods. Although this may be expected from the uniformity of the dietary composition and lack of variation posed by concentrate-based meals, this provides important supportive information for the design and justification of future trials. The stability in these ponies presents evidence on the sampling frequency that may be required to capture sufficient representation of the bacterial population in the faeces.

## Conclusion

This is the first study to investigate the stability of a faecal microbial population with such high intensity of sampling over two 72h periods, both in terms of molecular and metabolomics profiling. This has provided evidence of strong pony-specific bacterial populations that are stable over 72h, as are the changes in bacterial population and metabolome between the trial periods. This presents important evidence to support and guide the construction and sampling frequency of future animal trials.

## Materials and Methods

All procedures were conducted in accordance with Home Office requirements, and were approved by the University of Liverpool Animal Welfare Committee and the Faculty of Veterinary Science Research Ethics Committee. Permission for these ponies to be used in the trial was obtained from the owner.

### Details of Ponies and Faecal Sample Collection

Six Welsh Mountain pony mares (age 10 ±2 years, outset body mass 246 ±20 kg), which had previously been kept out on pasture, were obtained at least two weeks prior to the study. They were stabled in individual pens on wood shavings and had been allowed to adapt to SPILLERS Happy Hoof: comprising 25% crude fibre (CF), 8% crude protein (CP), 9.5% ash, 4–5% simple sugars, 5% starch, gross energy (GE) 16.9 MJ/kg dry matter (DM), fibre length 1–3 cm (HH) during the 7 days prior to the experiment starting, as described previously [34], [35]. Thereafter they were fed *ad libitum* on HH for the duration of the experiment (12 weeks). One week into the experiment, all defecations were collected over a 72h trial period (TP1) and the time of defecation was recorded. This was repeated 11 weeks later (TP2). All samples were frozen at –20°C immediately post-defecation and a sub-sample used for analysis. Two-way ANOVA was used to compare defecation number (GenStat, 13^th^ Edition).

### Pilot study to test stability levels

A preliminary investigation was performed to assay the extent, if any, of the specific host animal on the bacterial stability levels and the sampling frequency depth by using all defecations obtained during TP2 from ponies 2 and 5. These 2 ponies were selected for comparison due to them having an identical number of defecations (n = 37) over the 72 h. TP2 was used due to the greater period of time that the ponies had been maintained on the same management and diet. Pony specific clustering was identified (Figure S1) in the pilot study and a more comprehensive study, involving samples from all animals, and from both trial periods, was performed.

### Sample Preparation for DNA extraction

Ten regularly spaced faecal samples (mean interval 6.5 hours + 1.65) from each pony and trial period (n =  120) were freeze-dried for 48 hours and then bead beaten for 30 seconds at 5000rpm (maximum speed) in a Minibeadbeater™ (Biospec products Inc., Bartlesville, Oklahoma) and stored at –20°C. DNA was then extracted using QIAGEN QIAamp® DNA stool mini kits (Qiagen Ltd., UK) with an increased initial lysis incubation temperature of 95°C, as recommended by the manufacturers [3]. Physical disruption (via bead beating) and increased lysis temperature were to prevent any extraction bias due to the assumed presence of Gram positive bacteria.

### PCR Conditions for amplification of the 16S *rRNA* gene

PCR was performed using a bacterial-specific primer pair for the 16S *rRNA* gene, unlabelled 27F (5′-AGA GTT TGA TCC TGG CTC AG-3′) and cyanine labelled 1389R (5′-ACG GGC GGT GTG TAC AAG-3′) [36]. Amplification was performed using a BIORAD MyCycler™ thermal cycler with the following program: an initial 4 min denaturation at 94°C followed by 25 cycles of 1 min denaturation at 94°C, 1 min annealing at 55°C and 1 min extension at 72°C. A final cycle of 1 min at 94°C, 1 min at 55°C and elongation for 5 min at 72°C completed the PCR. The reaction cocktail used throughout was: 0.1 U/µl GoTaq Flexi DNA polymerase (Promega); 1x reaction buffer (Promega); 1.75 mM MgCl_2_; 0.5 µM of each primer; and 0.2 mM of each of the dNTPs with 150 ng of template DNA. DNA concentration was quantified by Nanodrop (ND-1000 spectrophotometer). All reactions were carried out in a final volume of 25 µl.

### Restriction Enzyme Digestion of 16 *rRNA* Amplicons

The PCR product was purified (Millipore MultiScreen® PCR_µ96_ plate with 20 inches Hg vacuum) and the DNA concentration for each sample was determined by spectrophotometry (Nanodrop® ND-1000 spectrophotometer). Restriction enzyme digestion was performed using 0.1 U/µl HaeIII with 150 ng DNA; 0.1 U/µl ScrFI with 125 ng DNA and 0.2 U/µl MspI with 250 ng DNA (all enzymes purchased from New England Biolabs) with their respective recommended buffers and reaction condition in 50 µl volumes with single enzyme digestions for 5 hours.

The purified restriction digest products were re-suspended in sample loading solution with DNA Size Standard 600 (Beckman Coulter Inc., Fullerton, USA). Size fractionation was performed on a CEQ™ 8000 Beckman Coulter DNA sequencer, with Local Southern Algorithm and calibrated using AE version 2 (manufacturer's recommendation, CEQ™ 8000 Beckman Coulter software) in the CEQ™ 8000 software. Data were analysed when exported with a slope threshold of 10 and a relative peak height of 10% within the CEQ™ 8000 software. Data were binned (1bp in size) and exported using the AFLP (amplified fragment length polymorphism) facility in the CEQ™ 8000 software. Peaks that lay outside the detected range of the size standard across all samples were removed. Relative peak height was calculated for each sample prior to further analysis [3].

### Analysis and determining similarity levels between restriction fragment patterns

Terminal Restriction Fragment Length Polymorphism (TRFLP) data for all 3 restriction enzymes for ten samples per pony for each of the trial periods were combined by stacking the data and analysed together. This was done due to the differences in restriction sites between enzymes, each with the potential to differentiate between differing species, without which data interpretation are limited [37], [38]. Data were, therefore, combined to generate a better estimation of the similarity of the population structure within the samples and limiting the effect of a conserved terminal restriction site within populations.

### Determination of short chain fatty acid concentration

A faecal slurry was made as 20% w/v with frozen faeces and distilled water. The slurry was homogenised in a stomacher for 1 minute (Stomacher ‘80’ biomaster). Four ml of the slurry was added to 1 ml orthophosphoric acid (with an internal standard of 20 mM 2-ethyl butyric acid) and vortexed. Samples were allowed to precipitate for at 24 h. Two millilitres were then taken and syringe-filtered through a glass fibre prefilter (Millipore, USA) and a nitrocellulose membrane (0.65 µm pore, Millipore, USA) into a glass vial and capped. SCFAs (short chain fatty acids) were detected by gas chromatography using Varian CP3380 with the HP-FFAP column from J&W Scientific (10 m length ×0.53 mm I.D. × 1 µm film thickness). The temperature was 80°C initial temperature, 1 minute hold after injection and then 20°C/minute up to 120°C, 6.2°C/minute up to 140°C, 20°C/minute up to 205°C with final hold for 5.52 minutes. The flame ionization detector and injector were at 250°C. One microlitre was injected using helium as the carrier gas at 6psi. SCFA concentrations were then determined relative to reference standards (8 mM SCFA/4 mM 2-ethylbutyric acid; 5 mM SCFA/10 mM 2-ethylbutyric acid; 5 mM SCFA/4 mM 2-ethylbutyric acid and 4 mM SCFA/4 mM 2-ethylbutyric acid).

### Fourier Transform Infrared (FTIR) Spectrophotometry analysis

Freeze dried and bead beaten faecal samples (100 mg) were re-suspended with 1 ml of distilled water and transferred to an FTIR 96 sample plate. Samples were analysed in triplicate over different plates and all samples were dried at 40°C before FTIR analysis using a Bruker Optics Tensor 27 with HTS-XT for 96 well plate reading. Spectra in the range of 4000–600 cm^−1^ were compared with any replicate outliers removed. Replicates were then averaged and spectra were normalised by conversion to relative values across samples to minimise loading variation. PCA and DFA with cross validation were then used to test for spectral clustering by pony (Matlab R2009a).

### Statistical Analysis

Analysis of Terminal Restriction Fragment (TRF) samples was performed by Hamming Distances (for binary, presence/absence data) and Manhattan Distances (for relative abundance) coupled with Unweighted Pair Group Method with Arithmetic mean (UPGMA); Principal Component Analysis (PCA); and Discriminant Function Analysis (DFA). UPGMA analysis was performed using Neighbor within the PHYLIP [39] suite of programs. Permutation multivariate analysis of variance (PERMANOVA) was performed using PERMANOVA+ (version 1.0.2; primer-E, Ivybridge, UK). The fourth root transformation was used on relative abundance data with 9999 unrestricted permutations [3], [40]. 9999 unrestricted permutations were also performed on the Hamming Distances (binary data). PCA and DFA were performed using MATLAB (R2009a) software. DFA was cross validated by splitting the data to training and test data in a 2:1 ratio. Diversity measures of Shannon Diversity and Evenness Indices [41], number of TRF and SCFA concentrations were compared by two-way ANOVA with Bonferroni *post hoc* correction used for multiple comparisons used (GENSTAT, 13^th^ Edition).

## Supporting Information

Figure S1 Dendrogram of Manhattan Distances from all defections from pony 2 and pony 5 from trial period 2.
(TIF)

Table S1 Ratio of each major SCFA, for each pony and trial period.
(DOCX)
