# Supplementary figures and images for: Strong Stability and Host Specific Bacterial Community in Faeces of Ponies
Source: PLoS One. 2013 Sep 11;8(9):e75079. doi: 10.1371/journal.pone.0075079 (PMC3770578; doi:10.1371/journal.pone.0075079)

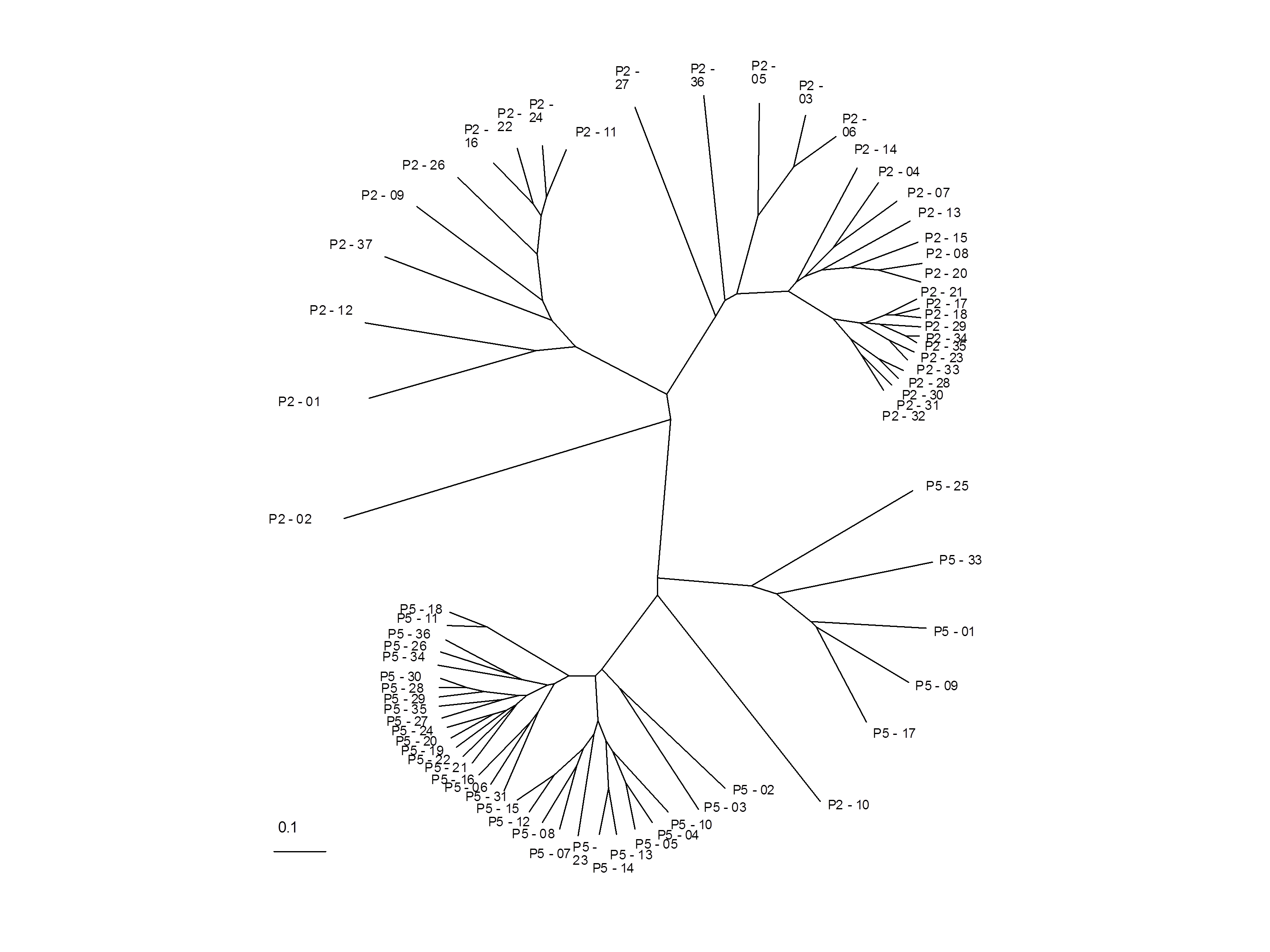

Supplement: Figure S1 — Dendrogram of Manhattan Distances from all defections from pony 2 and pony 5 from trial period 2. (TIF) [file pone.0075079.s001.tif]
