# Supplementary material for: Strong Stability and Host Specific Bacterial Community in Faeces of Ponies
Source: PLoS One. 2013 Sep 11;8(9):e75079. doi: 10.1371/journal.pone.0075079 (PMC3770578; doi:10.1371/journal.pone.0075079)
Supplement: Table S1 — Ratio of each major SCFA, for each pony and trial period. (DOCX) [file pone.0075079.s002.docx]

**Table S1 – Ratio of each major SCFA, for each pony and trial period.**

|  | Pony | | 1 | 2 | 3 | 4 | 5 | 6 | P^2^ value |
| --- | --- | --- | --- | --- | --- | --- | --- | --- | --- |
| Acetate | TP1 | Mean | 81.05 | 80.76 | 79.91 | 81.90 | 78.18 | 80.87 | * |
|  |  | SE | 0.675 | 0.445 | 0.388 | 0.583 | 1.184 | 1.143 |  |
|  | TP2 | Mean | 81.79 | 80.04 | 80.87 | 84.11 | 83.76 | 81.20 | * |
|  |  | SE | 1.167 | 0.807 | 0.920 | 1.120 | 1.045 | 0.843 |  |
|  | P^1^ value | | ns | ns | ns | ns | ** | ns |  |
| Propionate | TP1 | Mean | 14.02 | 13.49 | 12.39 | 13.44 | 14.64 | 12.05 | ns |
|  |  | SE | 0.506 | 0.371 | 0.299 | 0.554 | 0.954 | 0.976 |  |
|  | TP2 | Mean | 11.51 | 15.45 | 12.20 | 11.04 | 10.59 | 12.44 | *** |
|  |  | SE | 0.860 | 0.607 | 0.660 | 0.798 | 0.557 | 0.789 |  |
|  | P^1^ value | | * | ** | ns | * | ** | ns |  |
| Butyrate | TP1 | Mean | 4.23 | 4.73 | 5.63 | 4.39 | 5.51 | 5.27 | *** |
|  |  | SE | 0.142 | 0.155 | 0.110 | 0.151 | 0.255 | 0.298 |  |
|  | TP2 | Mean | 5.26 | 3.89 | 5.33 | 4.04 | 4.23 | 5.19 | *** |
|  |  | SE | 0.288 | 0.155 | 0.258 | 0.312 | 0.378 | 0.229 |  |
|  | P^1^ value | | ** | ** | ns | ns | ** | ns |  |
| Valerate | TP1 | Mean | 0.69 | 1.02 | 2.06 | 0.28 | 1.67 | 1.82 | *** |
|  |  | SE | 0.096 | 0.093 | 0.071 | 0.150 | 0.184 | 0.214 |  |
|  | TP2 | Mean | 1.44 | 0.62 | 1.60 | 0.81 | 1.42 | 1.16 | ** |
|  |  | SE | 0.242 | 0.071 | 0.088 | 0.121 | 0.287 | 0.210 |  |
|  | P^1^ value | | ** | ** | *** | ** | ns | * |  |

P^1^ values identify significant changes between TP within pony and each SCFA. P^2^ values identify significant differences between ponies, within TP and each SCFA. ns – not significant, * P<0.05; ** P<0.01; *** P<0.001
